# Cohort Profile: The China Severe Trauma Cohort (CSTC)

**DOI:** 10.2188/jea.JE20220290

**Published:** 2024-01-05

**Authors:** Yao Yang, Minlan Yuan, Yu Zeng, Yuanjing Xie, Yueyao Xu, Dengbin Liao, Yongmei Chen, Meiru Chen, Yuanyuan Qu, Yao Hu, Wei Zhang, Huan Song

**Affiliations:** 1 West China Biomedical Big Data Center, West China Hospital, Sichuan University, Chengdu, China; 2 Med-X Center for Informatics, Sichuan University, Chengdu, China; 3 Mental Health Center, West China Hospital of Sichuan University, Chengdu, China; 4 Shaanxi University of Chinese Medicine, Xian, China; 5 Department of Orthopaedics and Trauma Center, West China Hospital, Sichuan University, Chengdu, China

**Keywords:** cohort, physical trauma, post-trauma stress reaction, psychopathology, genetics

## Abstract

**Background:**

We sought to establish a prospective hospital-based cohort, featuring detailed multidimensional data of trauma patients with active follow-ups, which can be a reliable data source for all studies focusing on the effects or underlying mechanistic pathways of environmental and biological factors on multiple interested trauma-related outcomes, particularly the incidence and trajectory of trauma-related psychopathology, in a Chinese population.

**Methods:**

The China Severe Trauma Cohort (CSTC) enrolled all traumatized individuals aged 12 to 80 years admitted to the Trauma Center of West China Hospital between March 1^st^, 2020 and July 8^th^, 2022. The bio-sample and detailed questionnaire data were collected at recruitment, and phone/internet follow-ups were scheduled at 1, 3, 6, and 12 months after the baseline. Long-term health outcomes are planned to be obtained from administrative databases through data linkage.

**Results:**

A total of 2,500 trauma patients were enrolled (response rate = 87.1%) with an average age of 46.01 years, and most of the participants were males (62.6%). The proportions of participants with blood and fecal sample collected at baseline were 93.8% and 66.3%, respectively. As of August 31^st^, 2022, the follow-up rate was 90.0%, 77.0%, 76.5%, and 89.0% for 1-, 3-, 6-, and 12-month follow-ups, respectively. Fall/wrench (47.6%) and traffic accident (26.2%) were the top causes of current trauma. The most common psychopathology at recruitment was sleep disturbance (39.4%), followed by depression (22.6%), anxiety (18.2%), and acute stress reaction (7.8%), all of which showed recovering trajectories during the follow-up period, particularly the first 3 months after baseline.

**Conclusion:**

CSTC provides a platform with multidimensional data to study both short-term and long-term trauma-related health consequences, prompting early identification and intervention for individuals with high risk of health decline after trauma exposures.

## INTRODUCTION

Severe trauma, mainly referring to injuries caused by accidents, drowning, violence, and natural disaster, such as high-magnitude earthquake, is the primary cause of disability^1^^,^^2^ and the sixth-leading cause of mortality worldwide.^3^ In China, it is estimated that at least 60 million cases of severe trauma with requirement of emergency treatment, as well as approximately 1 million trauma-related disabilities or deaths, happened every year.^4^ Given the astonishing proportion of the affected population and substantial social and disease burden induced by such a condition, it is important to improve the medical management of trauma through enhancing the trauma care system and developing more efficient and effective interventions for achieving better prognosis. Following the guidelines proposed by World Health Organization in 2012,^5^ existing efforts in China include the establishment of the China Trauma Rescue & Treatment Association^6^ and National Center for Trauma Medicine in 2016 and 2019, respectively, which aim to prompt essential trauma care by optimizing the process of trauma care and its quality, as well as constructing qualified trauma care centers in more than 700 counties.^3^

As a Chinese southwestern province located on a vibrant seismic belt, Sichuan faces high possibility of having catastrophic natural disasters, such as the Wenchuan Earthquake in 2008. Also, along with a rapid increase in use of motor vehicles^7^ and corresponding increase in traffic accidents, this place has been ranked as one of the provinces with highest prevalence of acquired disability in China,^8^ with annual number of injury cases increasing from 21,257 to 44,112 from 2006 to 2015.^9^ Therefore, we established the China Severe Trauma Cohort (CSTC) based on the Trauma Center of West China Hospital,^10^ which involved patients with sudden and serious injuries that require continuous hospital utilization after emergency care. This trauma center has more than 100 beds and preform approxiately 1,900 trauma-related operations annually.

In addition to the high morbidity and mortality, the psychological impacts of severe traumatic events, which present as various psychiatric symptoms and stress-related disorders, including acute stress disorder (ASD) and posttraumatic stress disorder (PTSD),^11^ have gained increasing attentions from the public and researchers. The new-onset symptoms of depression and anxiety are reported to be 12.6–23.6%,^12^^,^^13^ and a systematic analysis of adult road traffic crash survivors in 35 studies summarized an incidence of PTSD that varied from 8.0–45.0%, indicating great geographic variations.^14^ Further, psychiatric morbidity occurred after trauma may delay the recovery of patients’ injury,^15^ increases possibility of having impaired physical and social function,^16^ and consequently deteriorated life quality^17^^,^^18^ among traumatized population. Importantly, the potential links between trauma-related psychiatric disorders and future elevated risk of various somatic diseases in the long run have also been revealed in recent investigations conducted by our group^19^^–^^21^ and others,^22^^,^^23^ highlighting the importance of developing effective interventions for early prevention of psychopathology, to interrupt both mental and physical health decline after trauma exposure. Nevertheless, due to the lack of data, studies assessing the psychiatric changes among traumatized population in China are currently absent.

Therefore, we established the China Severe Trauma Cohort (CSTC) based on the Trauma Center of West China Hospital, which involved patients with sudden and serious injuries that require continuous hospital utilization after emergency care. The overarching aim of CSTC is to provide a reliable data source with detailed multidimensional data (eg, trauma experience, lifestyle factors, somatic and psychiatric history, process of medical management, laboratory biomarkers, and genetics) of traumatized individuals, a wide range of trauma-related outcomes measured shortly after the trauma and during 1 year of follow-up, as well as long-term health consequences derived from linked administrative registers. The CSTC database is to support all studies exploring the complex interplays of environmental and genetic components on interested trauma-related outcomes, and in the current research, we made a detailed description regarding CSTC establishment, baseline and follow-up characteristics of CSTC, and the strength and limitation of CSTC.

## MAIN FEATURES

Based on West China Trauma Center, the pilot study was conducted between April 1^st^, 2020, and May 31^st^, 2020 to develop standardized pipelines for data and sample collection. Then, the CSTC study was officially launched on June 1^st^, 2020, with the purpose of recruiting 3,000 patients aged between 12 and 80 years with an experience of severe trauma within 3 months.

**Figure 1.**
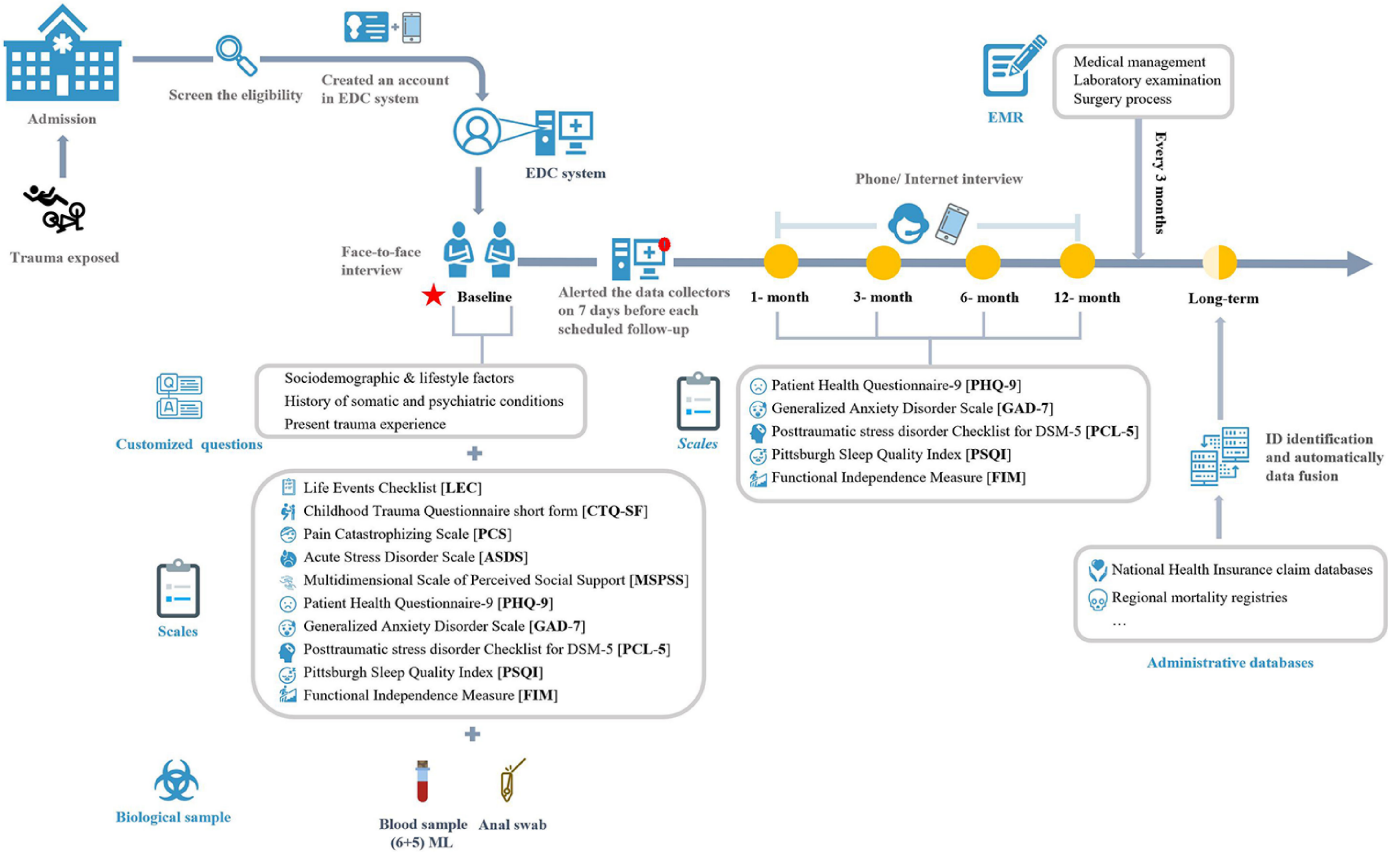
Study design

**Figure 2.**
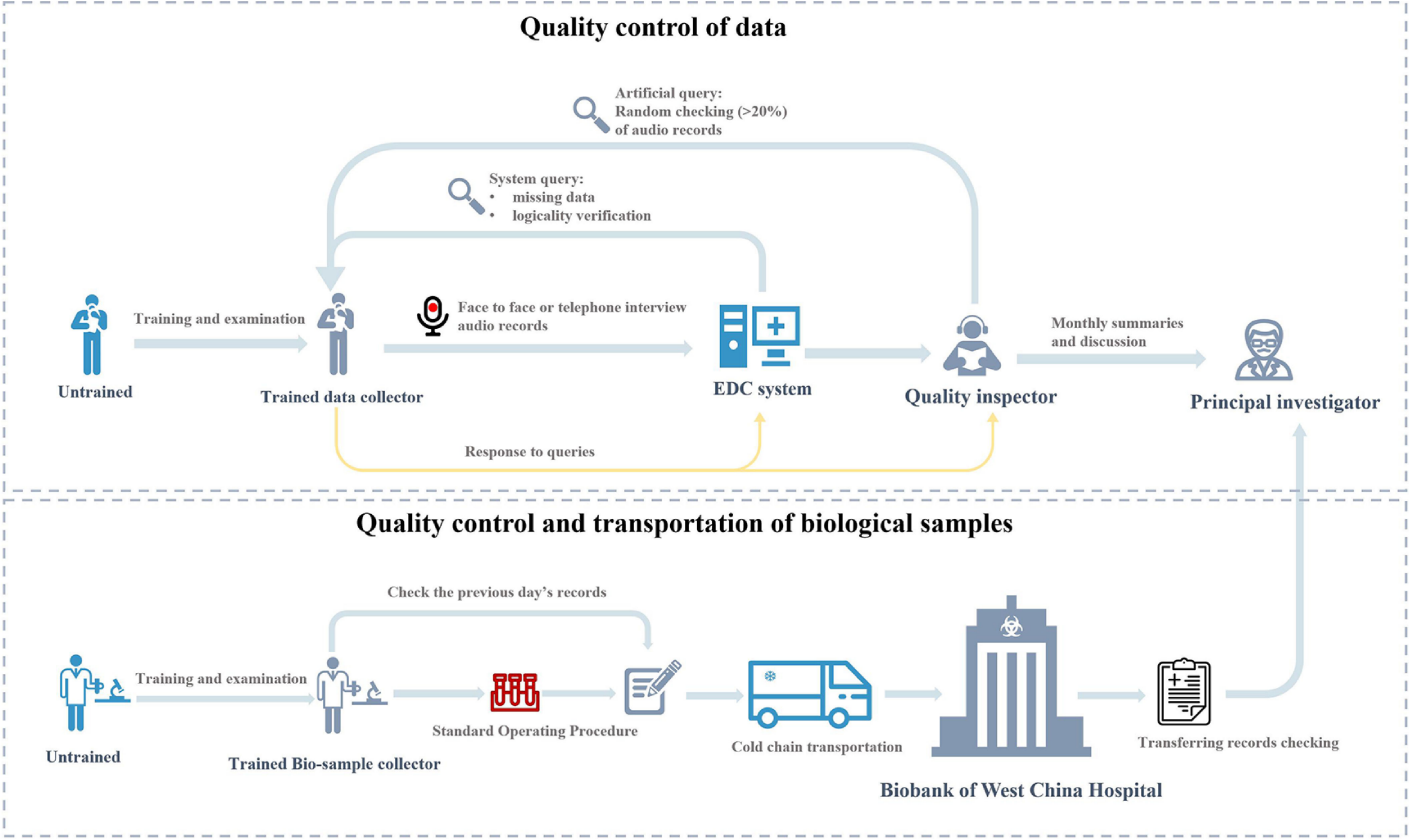
Quality control process

This prospective cohort study is featured by bio-sample and detailed data collection both at recruitment and at several follow-up points (ie, 1, 3, 6, and 12 months after the enrollment; Figure 1), as well as multiple approaches for stringent quality control (Figure 2). In addition, we plan to obtain long-term health related outcomes (eg, diagnosis of multiple diseases and deaths) through data linkage with administrative databases (eg, National Health Insurance claim databases and regional mortality registries). All data and sample collections were done by well-trained and full-time data collectors. The baseline survey was performed through face-to-face interview using touchscreen questionnaires. Originally, the 1-month follow-up was planned to be in person as well, with repeat biological sample collection. However, due to the local outbreak of COVID-19, it was then (on December 1^st^, 2020) switched to phone/internet interviews, just as the follow-ups at 3, 6, and 12 months after the enrollment, for maintaining the feasibility. Notably, participants who failed to complete one planned follow-up would still be contacted for the next follow-ups, unless they requested to stop future communications or withdraw their inform consent.

This cohort study is supported by the National Natural Science Foundation of China and 1.3.5 project for disciplines of excellence, West China Hospital, Sichuan University. The study protocol was approved by the Biomedical Research Ethics Committee of West China Hospital in Sichuan University (2020.243).

## PARTICIPANTS

All participants were recruited from Trauma Center of West-China Hospital. Participants that considered eligible were: (i) injury patients with trauma experience within 3 months who were admitted to this trauma center for the first time (patients who have received medical care in their local hospital were also included); (ii) 12–80 years old; (iii) capable of understanding the content of questionnaires; (iv) residents of Sichuan Province. We further excluded those: (i) having any structural abnormality of brain according to Computed Tomography (CT) scan in attendance (eg, brain contusion, intracranial hematoma, depressed or comminuted fractures, diffuse axonal injury, and cerebral hernia); (ii) exposed to persistent amnesia more than 24 hours after their trauma; (iii) failed to complete questionnaires or biological sample collection due to unconsciousness or fatal conditions. All of the participants signed an informed consent form prior to data collection.

## OUTCOMES AND FOLLOW-UP

For patient requirement at baseline (Figure 1), the eligibility of newly admitted patients in the Trauma Center of West-China Hospital were screened daily. Then, the trained data collectors created a new account for each eligible patient in the electronic data collection (EDC) system, based on the patient’s ID number and phone number. After signing the informed consent, research nurses in the department collected the blood and fecal sample according to the study protocol; and face-to-face interviews were scheduled during day time in the ward, where the trained data collectors asked about information about sociodemographic and lifestyle factors, experience of traumatic events (during childhood and lifetime), detailed trauma experience that led to this inpatient admission, somatic fitness, social supports, and psychiatric symptoms (ie, anxiety, depression, stress reaction, and sleep quality) using customized questions and a series of well-validated scales in touchscreen questionnaires (see details in the ‘survey instruments’ below).

The EDC system alerted the data collectors on 7 days before each scheduled follow-up (1, 3, 6, and 12 months after the enrollment), which allowed a variety of actual follow-up date for a week. We collected information on psychiatric (ie, symptoms of anxiety, depression, stress reaction, and sleep quality) and somatic conditions (ie, wound healing, limitation of moderate physical activity or walking up and down the stairs, functional independence) at each follow-up point, using the same questions or scales as the baseline survey. Further, we matched the specific ID of each patient in administrative database to obtain the details of medical or death records for long term.

The quality of both baseline and follow-up data was guaranteed by the logical quality checks and extreme-value inquiries that implemented in the EDC systems, as well as a random checking (>20%) of audio records of the whole interview process by a quality inspector (Figure 2). Any disagreements were doubt checked by data collectors and quality inspector together; and the quality inspector made monthly summaries on progress of data collection and measures of data quality, for a regular discussion with the principal investigator.

Every 3 months, we additionally linked the data of EDC system to electronic medical records (EMR) in the hospitals, for obtaining detailed information on medical management, laboratory examination results, surgery process (if any), during the whole study period for all included participants. For obtaining health related consequences after 1 year of recruitment, health records derived from the administrative databases with full coverage of study participants will be linked once per year.

## MEASUREMENT

### Survey instruments

Sociodemographic and lifestyle factors, including age, gender, ethnics (Han or other), body weight (kg), height (m), education level (elementary school and below, middle school, senior high school or technical secondary school, college, or postgraduate and above), living area (rural or city), current smoking status (yes or no), current alcohol consumption (yes or no), monthly household income (≤3,000, 3,001–6,000, 6,001–9,000, 9,001–12,000, or >12,000 RMB), self-evaluated economic status (lower than average, average, or above the average), occupation status (full-time, part-time, unemployed, house-wife or house-husband, retired, or student), marital status (married, unmarried, divorced or widowed) were collected using customized questions. We additionally asked information about history of severe somatic diseases (see eTable 1), as well as the history of psychiatric disorders (yes or no), and psychoactive substance dependence (yes or no) before this current trauma experience. We also collected detailed data on prior exposure to traumatic events (using Life Events Checklist [LEC]^24^ and Childhood Trauma Questionnaire [CTQ-SF]^25^) and pain experience (using a single question ‘have you ever have chronic pain’ and using the Pain Catastrophizing Scale [PCS]^26^).

Questions related to details of the present trauma experience which led to the Trauma Center admission contained 18 items, including time of injury (hour and date), time passed since injury (days or months), the exact time of injury, the cause of injure (traffic accident, explosion, high fall, hitting by objectives, machine related accident, fall/wrench, cutting, or other), family/intimate friends were injured (yes or no) or died (yes or no), witnessing family/intimate friends/strangers’ injure (yes or no) or death (yes or no), strangers were injured or died (yes or no), witnessing stranger’s injure or death (yes or no), feeling fear during the event (yes or no, scaled from 0 to 7 if yes), self-evaluated severity of present injury (scaled from 0 to 5), ever loss of consciousness (yes or no, and the duration if yes), as well as the extend of current pain (measured by the distance on the 10-cm line between the “no pain” anchor and “worst pain”), function impairment (ie, the limitation of moderate physical activity [greatly, somehow, or never] and walking up/down stairs [greatly, somehow, or never]) and independence (using the Functional Independence Measure [FIM],^27^ which comprises 13 motor items and 5 cognitive items with a total scores range of 13–126, to evaluate the functional independence with a higher score on behalf of better independence).

We assessed the presence of psychopathology after trauma using well-validated scales, including the 9-item Patient Health Questionnaire-9 (PHQ9) for depressive symptoms,^28^ the 7-item Generalized Anxiety Disorder Scale (GAD-7)^29^ for anxious symptoms, and 19-item Acute Stress Disorder Scale (ASDS; for baseline)^30^ or a 20-items Checklist for DSM-5 (PCL-5, for both baseline and follow-ups)^31^ for severe stress reactions. Sleep quality was measured using Pittsburgh Sleep Quality Index (PSQI),^32^ a 19-item self-report questionnaire evaluating seven dimensions of sleep (ie, subjective sleep quality, sleep latency, sleep duration, habitual sleep efficiency, sleep disturbances, use of sleep medications, and daytime dysfunction) during the past week. In addition, we evaluated the perceptions of social support from family or friends using a 12-items Multidimensional Scale of Perceived Social Support (MSPSS).^33^

All details of these questions and scales, together with their cutoff points and reported measures of validity among Chinese population could be found in eTable 2.

### Biological sample collection and storage

Blood and fecal samples were collected within 24 hours after the recruitment (Figure 1). We additionally re-collected these biological samples at the 1-month follow-up for 211 participants, before the change of study protocol.

For each participant, a total of 11 mL venous blood sample was extracted by research nurses, with 6 mL in an ethylenediaminetetraacetic acid (EDTA) tube and 5 mL in a serum separator tube. The handling of the blood samples, within 2 hours after the sample collection, included centrifugation (3,000 RPM, 4°C, 15 min for obtaining serum, plasma and red blood cells), aliquoting to 12 1.5 mL freezing tubes (serum, plasma, and blood cells each in 4 freezing tubes which temporally stored in −20°C refrigerators in Trauma Center), and documentation using barcode systems (Logene Software co., LTD, Wuxi, China). In addition, we used anal swabs (Shanghai mixeobio co., LTD, Shanghai, China) to collect feces of our participants after fully informed. The nurses wet the anal swab using isotonic saline, and rotated the swab twice into participant’s rectum with a depth about 3∼4 cm. Then swabs were kept in 1.5 mL microtubes and stored at −20°C for temporary preservation.

All samples were processed by well-trained data collectors who were required to strictly follow the standard operating procedure with time of process and temperature of refrigerator recorded. These samples were then periodically (every 2–4 weeks) transferred by a cold chain transportation company to a centralized biobank of the West China Hospital, Sichuan University and stored at −80°C in cryogenic refrigerators (Figure 2). After samples were well stored in the biobank, the quality inspector filled in the transportation form for tracing the transfer process, and double checked the accuracy of all information (including basic information about the patients and the accurate storage place) related to samples, together with a biobank staff.

## BASELINE AND FOLLOW-UP CHARACTERISTICS

Overall, upon August 31^th^, 2022, among 2,872 eligible patients, a total of 2,500 were successfully enrolled in our cohort, corresponding to a response rate of 87.1% (2,500/2,872) at baseline. The major reasons for nonparticipation included detected structural abnormality of brain CT scan after recruitment (37.6%, 140/372), refusal to join (19.1%, 71/372), and could not complete the questionnaires due to unconsciousness or having severe somatic diseases (13.4%, 50/372). The proportions of participants with blood and fecal sample at baseline were 93.8% (2,346/2,500) and 66.3% (1,657/2,500), respectively. Further, 211 participants had repeat blood samples from both baseline and 1-month follow-up.

The completeness of follow-ups for the recruited participants were 90.0% (2,169/2,410), 77.0% (1,705/2,213), 76.5% (1,469/1,920), and 89.0% (1,235/1,387) at 1, 3, 6, and 12 months after the recruitment, respectively. We observed 14 deaths during the 1-year active follow-up, and the major reasons for the loss of follow-up were unwillingness to answer follow-up questions (86.7%, 124/143) and not available after repeat contacts (9.1%, 13/143).

The average age of CSTC participants was 46.01 years (standard deviation [SD], 16.47) and most of them were male (62.6%, 1,564/2,500; Table 1). Regarding the trauma experience, the participants, on average, experienced 2.55 (SD, 2.12) traumatic life events before this current trauma (eTable 3). The mean total CTQ-SF score (ie, measuring the childhood trauma experience) of participants was 34.14 (SD, 7.59), and mean scores for having emotional neglect, physical neglect, and emotional abuse were 9.26 (SD, 3.72), 8.58 (SD, 3.37), and 5.85 (SD, 1.74), respectively. For current trauma leading to Trauma Center admission, 91.6% (2,290/2,500) participants had the trauma within 2 weeks before the recruitment (Table 2). Fall/wrench (47.6%, 1,190/2,500) and traffic accident (26.2%, 654/2,500) were the most common trauma causes, followed by high fall (8.0%, 199/2,500), hitting by objects (5.4%, 135/2,500), cutting (5.2%, 129/2,500), machine-related accident (3.2%, 79/2,500), explosion (0.2%, 4/2,500), and others (3.9%, 98/2,500). We further presented the sociodemographic characteristics and history of somatic and psychiatric conditions of all CSTC participants, as well as by cause of trauma (ie, fall/wrench, traffic accident, and others), in Table 1 and eTable 3. In brief, with similar proportion of the ‘married’ status, patients who injured due to fall/wrench were more likely to be older, female, living in rural area, not current smoker or alcohol drinker, having a history of somatic diseases and chronic pain, but having higher socioeconomic status (eg, higher proportions of having college and above educational attainment, monthly income, and perceived economic status), compared to participants with other trauma causes. Also, compared to participants with other causes of trauma, patients who were injured in traffic accidents reported a higher possibility of experiencing severe psychological trauma, representing as more participants with feeling of fear, having family/intimate friends/strangers injured or died during the event, although the extent of movement limitations (ie, having limitation of moderate physical activity or walking up/down stairs) seemed comparable between these subgroups (Table 2).

**Table 1.** Basic characters of study participants, overall and by different cause of trauma

Variable	Total^a^ *N* = 2,500	By cause of trauma		

		fall/wrench *N* = 1,190	traffic accident *N* = 654	other *N* = 644
Male, *n* (%)	1,564 (62.56)	623 (52.35)	406 (62.08)	531 (82.45)
Age, mean (SD), year	46.01 (16.47)	49.18 (17.61)	44.44 (15.38)	41.72 (14.05)
BMI, mean (SD), kg/m^2^	23.36 (3.46)	23.41 (3.48)	23.48 (3.55)	23.14 (3.34)
18.5	182 (7.28)	84 (7.06)	53 (8.10)	44 (6.83)
18.5–25	1,515 (60.60)	722 (60.67)	380 (58.10)	412 (63.98)
25–30	645 (25.80)	316 (26.55)	174 (26.61)	153 (23.76)
≥30	88 (3.52)	44 (3.70)	26 (3.98)	18 (2.80)
Unknown	70 (2.80)	24 (2.02)	21 (3.21)	17 (2.64)
Region, *n* (%)				
Urban	964 (38.56)	303 (25.46)	299 (45.72)	361 (56.06)
Rural	1,488 (59.52)	869 (73.03)	346 (52.91)	271 (42.08)
Unknown	48 (1.92)	18 (1.51)	9 (1.38)	12 (1.86)
Current smoking status,^b^ *n* (%)				
No	2,067 (82.68)	1,046 (87.90)	534 (81.65)	484 (75.16)
Yes	422 (16.88)	143 (12.02)	120 (18.35)	159 (24.69)
Unknown	11 (0.44)	1 (0.08)	0 (0.00)	1 (0.16)
Current alcohol consumption,^c^ *n* (%)				
No	2,063 (82.52)	1,021 (85.80)	542 (82.87)	497 (77.17)
Yes	427 (17.08)	168 (14.12)	112 (17.13)	147 (22.83)
Unknown	10 (0.40)	1 (0.08)	0 (0.00)	0 (0.00)
Marital status, *n* (%)				
Unmarried	469 (18.76)	200 (16.81)	129 (19.72)	139 (21.58)
Married	1,885 (75.40)	912 (76.64)	492 (75.23)	479 (74.38)
Divorced/Widowed	133 (5.32)	74 (6.22)	32 (4.89)	26 (4.04)
Unknown	13 (0.52)	4 (0.34)	1 (0.15)	0 (0.00)
Education, *n* (%)				
Elementary and lower	498 (19.92)	192 (16.13)	137 (20.95)	166 (25.78)
Junior school	753 (30.12)	287 (24.12)	219 (33.49)	244 (37.89)
Senior/Secondary school	611 (24.44)	323 (27.14)	162 (24.77)	124 (19.25)
Collage and above	511 (20.44)	331 (27.82)	95 (14.53)	82 (12.73)
Unknown	127 (5.08)	57 (4.79)	41 (6.27)	28 (4.35)
Occupation, *n* (%)				
Full-time	1,490 (59.60)	565 (47.48)	440 (67.28)	478 (74.22)
Part-time or unemployed^d^	554 (22.16)	270 (22.69)	138 (21.10)	145 (22.52)
Retired	456 (18.24)	355 (29.83)	76 (11.62)	21 (3.26)
Self-evaluated economic status, *n* (%)				
<average	372 (14.88)	149 (12.52)	115 (17.58)	108 (16.77)
average	1,836 (73.44)	900 (75.63)	455 (69.57)	472 (73.29)
>average	135 (5.40)	77 (6.47)	35 (5.35)	23 (3.57)
Unknown	157 (6.28)	64 (5.38)	49 (7.49)	41 (6.37)
Monthly household income (RMB), *n* (%)				
≤3,000	494 (19.76)	226 (18.99)	119 (18.20)	147 (22.83)
3,001–6,000	696 (27.84)	296 (24.87)	202 (30.89)	194 (30.12)
6,001–9,000	439 (17.56)	214 (17.98)	119 (18.20)	105 (16.30)
>9,000	643 (25.72)	365 (30.67)	140 (21.41)	136 (21.12)
Unknown	228 (9.12)	89 (7.48)	74 (11.31)	62 (9.63)
Perceived social support,^e^ *n* (%)				
Low supported	56 (2.24)	24 (2.02)	18 (2.75)	14 (2.17)
Moderate supported	361 (14.44)	150 (12.61)	107 (16.36)	103 (15.99)
High supported	1,732 (69.28)	850 (71.43)	430 (65.75)	450 (69.88)
Unknown	351 (14.04)	166 (13.95)	99 (15.14)	77 (11.96)

^a^12 participants with missing value of the trauma causes were not shown in these table.

^b^Participant chose “YES” in “Smoking” item was defined as current smoker.

^c^Participant chose “YES” in “Drinking alcohol” item was defined as current alcohol drinker.

^d^“unemployed” including the unemployed, housewife, house-husband, and student.

^e^Perceived social support was assessed by Multidimensional Scale of Perceived Social Support [MSPSS], with a total score 12–48 indicating low level of perceived social support, 49–68 indicating moderate level of perceived social support, and ≥69 indicating high level of perceived social support.

**Table 2.** Current trauma experience of study participants, overall and by different cause of trauma

Variable	Total^a^ *N* = 2,500	By cause of trauma		

		fall/wrench *N* = 1,190	traffic accident *N* = 654	other *N* = 644
Time passed since injury, *n* (%)				
≤2 days	1,016 (40.64)	530 (44.54)	212 (32.42)	270 (41.93)
3–7 days	985 (39.40)	455 (38.24)	295 (45.11)	229 (35.56)
8–14 days	289 (11.56)	129 (10.84)	83 (12.69)	76 (11.80)
15–30 days	163 (6.52)	60 (5.04)	48 (7.34)	54 (8.39)
>30 days	47 (1.88)	16 (1.34)	16 (2.45)	15 (2.33)
Injury cause, *n* (%)				
Traffic accident	654 (26.16)	—	654 (100.00)	0 (0.00)
Exploration	4 (0.16)	—	—	4 (0.62)
High fall	199 (7.96)	—	—	199 (30.90)
Hitting by objectives	135 (5.40)	—	—	135 (20.96)
Machine related accident	79 (3.16)	—	—	79 (12.27)
Fall/wrench	1,190 (47.60)	1,190 (100.00)	—	0 (0.00)
Cutting	129 (5.16)	—	—	129 (20.03)
Other	98 (3.92)	—	—	98 (15.22)
Unknown	12 (0.48)	—	—	0 (0.00)
Feeling fear during the event, *n* (%)				
No	1,493 (59.72)	774 (65.04)	340 (51.99)	375 (58.23)
Yes	1,005 (40.20)	415 (34.87)	314 (48.01)	268 (41.61)
Unknown	2 (0.08)	1 (0.08)	0 (0.00)	1 (0.16)
Scaled from 0–7 if ‘yes’, mean (SD)	4.87 (1.72)	4.38 (1.72)	5.43 (1.54)	4.96 (1.69)
Self-evaluated severity of present injury (scaled from 0–5), mean (SD)	3.82 (1.02)	3.65 (1.04)	4.02 (0.97)	3.92 (0.97)
Family/intimate friends were injured in present event, *n* (%)				
No	2,301 (92.04)	1,169 (98.24)	512 (78.29)	608 (94.41)
Yes	197 (7.88)	20 (1.68)	142 (21.71)	35 (5.43)
Unknown	2 (0.08)	1 (0.08)	0 (0.00)	1 (0.16)
If yes, witnessing family/intimate friends’ injury, *n* (%)				
No	78 (3.12)	6 (0.50)	56 (8.56)	16 (2.48)
Yes	118 (4.72)	14 (1.18)	86 (13.15)	18 (2.80)
Unknown	2,304 (92.16)	1,170 (98.32)	512 (78.29)	610 (94.72)
Family/intimate friends died in present event, *n* (%)				
No	2,475 (99.00)	1,188 (99.83)	640 (97.86)	635 (98.60)
Yes	23 (0.92)	0 (0.00)	14 (2.14)	9 (1.40)
Unknown	2 (0.08)	2 (0.17)	0 (0.00)	0 (0.00)
If yes, witnessing family/intimate friends’ death, *n* (%)				
No	12 (0.48)	0 (0.00)	7 (1.07)	5 (0.78)
Yes	11 (0.44)	0 (0.00)	7 (1.07)	4 (0.62)
Unknown	2,477 (99.08)	1,190 (100.00)	640 (97.86)	635 (98.60)
Strangers injured or died in present event, *n* (%)				
No	2,457 (98.28)	1,186 (99.66)	622 (95.11)	637 (98.91)
Yes	41 (1.64)	3 (0.25)	31 (4.74)	7 (1.09)
Unknown	2 (0.08)	1 (0.08)	1 (0.15)	0 (0.00)
If yes, witnessing strangers’ injury or death, *n* (%)				
No	22 (0.88)	3 (0.25)	16 (2.45)	3 (0.47)
Yes	19 (0.76)	0 (0.00)	15 (2.29)	4 (0.62)
Unknown	2,459 (98.36)	1,187 (99.75)	623 (95.26)	637 (98.91)
Having limitation of moderate physical activity, *n* (%)				
Never	2,055 (82.20)	972 (81.68)	562 (85.93)	512 (79.50)
Somehow	371 (14.84)	183 (15.38)	72 (11.01)	114 (17.70)
Greatly	72 (2.88)	34 (2.86)	20 (3.06)	18 (2.80)
Unknown	2 (0.08)	1 (0.08)	0 (0.00)	0 (0.00)
Having limitation of walking up/down stairs, *n* (%)				
Never	1,679 (67.16)	735 (61.76)	525 (80.28)	410 (63.66)
Somehow	182 (7.28)	95 (7.98)	50 (7.65)	37 (5.75)
Greatly	636 (25.44)	358 (30.08)	79 (12.08)	197 (30.59)
Unknown	3 (0.12)	2 (0.17)	0 (0.00)	0 (0.00)
Loss of consciousness in present event, *n* (%)				
No	2,312 (92.48)	1,166 (97.98)	547 (83.64)	595 (92.39)
Yes	178 (7.12)	22 (1.85)	107 (16.36)	49 (7.61)
Unknown	10 (0.40)	2 (0.17)	0 (0.00)	0 (0.00)

^a^12 participants with missing value of the trauma causes were not shown in this table.

We compared the baseline characteristics of the participants who completed the planned follow-ups with those who did not at 1 month (2,169/2,410 [90.0%] vs 241/2,410 [10.0%]) and 12 months (1,235/1,387 [89.0%] vs 152/1,387 [11.0%]) after the recruitment, and found those who failed to complete the follow-up questions tended to older and had higher proportions of choosing ‘unknown’ for most of the baseline questions (eTable 4).

**Figure 3.**
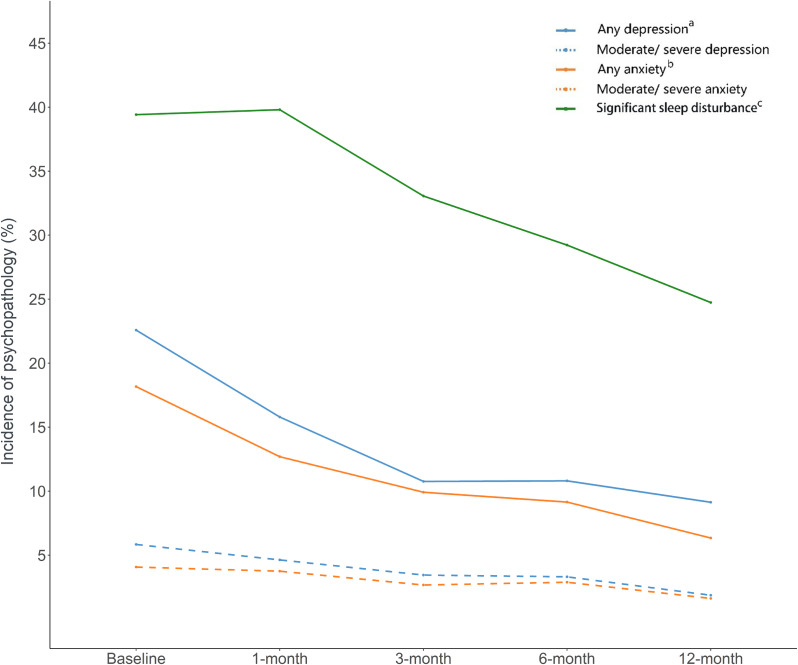
Incidence of psychopathology during the study period. ^a^Depression symptoms were measure by Patient Health Questionnaire-9 (PHQ-9), with a total score 5–9 indicating mild depression, and ≥10 for moderate/severe depression. ^b^Anxiety symptoms were measure by Generalized Anxiety Disorder Scale (GAD-7), with a total score 5–9 indicating mild anxiety, and ≥10 for moderate/severe anxiety. ^c^Significant sleep disturbance were determined by Pittsburgh Sleep Quality Index (PSQI) with a cut-off score >5.

As the incidence of psychiatric abnormalities after trauma was a major focus of this cohort, we summarize the presence of psychopathology, any or subtypes of psychiatric symptoms measured by corresponding scales, in Table 3 and eTable 5. At baseline, the most common psychopathology was sleep disturbance (39.4%, 1,003/2,499), followed by depression (22.6%, 205/908), anxiety (18.2%, 165/908), and acute stress reaction (7.8%, 195/2,499). Regarding the trajectories of psychopathology during the follow-ups, we generally observed a recovering curve for all studied psychiatric abnormalities (Figure 3)—the drops of incidence were most pronounced within 3 months after the recruitment and somewhat stable afterwards. For symptoms of PTSD specifically, we observed consistently low incidence (ie, determined by PCL-5, Table 3) among all participants (7.6% at baseline and 1.71% at the 12-month follow-up), although the rates were slightly higher for individuals with high perceived (ie, self-evaluated, 15.8% at baseline and 2.4% at the 12-month follow-up) or subjective (ie, having people injured or died during the event, 16.7% at baseline and 2.9% at the 12-month follow-up) trauma severity.

**Table 3.** Incidence of severe stress reaction among study participants

	Probably cases *n* (%)	Subscales, *n* (%)			

		Intrusion	Avoidance	Cognitions and mood	Arousal and reactivity
*For all participants*					
**Acute stress reaction^a^**	195/2,499 (7.80)	—	—	—	—
**Posttraumatic stress disorder^b^**					
Baseline	71/930 (7.63)	306/930 (32.90)	224/930 (24.09)	331/930 (35.59)	511/930 (54.95)
1-month follow-up	49/2,162 (2.27)	295/2,162 (13.64)	227/2,162 (10.50)	388/2,162 (17.95)	782/2,162 (36.17)
3-month follow-up	46/1,690 (2.72)	204/1,690 (12.07)	157/1,690 (9.29)	271/1,690 (16.04)	581/1,690 (34.38)
6-month follow-up	27/1,461 (1.85)	160/1,461 (10.95)	146/1,461 (9.99)	209/1,461 (14.31)	468/1,461 (32.03)
12-month follow-up	21/1,226 (1.71)	105/1,226 (8.56)	96/1,226 (7.83)	151/1,226 (12.32)	324/1,226 (26.43)
*Participants with self-evaluated current severity above average^c^*					
**Acute stress reaction**	97/750 (12.93)	—	—	—	—
**Posttraumatic stress disorder**					
Baseline	36/228 (15.79)	96/228 (42.11)	77/228 (33.77)	106/228 (46.49)	155/228 (67.98)
1-month follow-up	24/643 (3.73)	109/643 (16.95)	75/643 (11.66)	143/643 (22.24)	254/643 (39.50)
3-month follow-up	19/495 (3.84)	70/495 (14.14)	50/495 (10.10)	99/495 (20.00)	198/495 (40.00)
6-month follow-up	12/478 (2.51)	66/478 (13.81)	71/478 (14.85)	85/478 (17.78)	186/478 (38.91)
12-month follow-up	10/423 (2.36)	46/423 (10.87)	49/423 (11.58)	58/423 (13.71)	142/423 (33.57)
*Participants experienced family/* intimate friends */* strangers *died/injured in current event*					
**Acute stress reaction**	38/235 (16.17)	—	—	—	—
**Posttraumatic stress disorder**					
Baseline	16/96 (16.67)	41/96 (42.71)	39/96 (40.62)	49/96 (51.04)	60/96 (62.50)
1-month follow-up	7/203 (3.45)	40/203 (19.70)	32/203 (15.76)	57/203 (28.08)	83/203 (40.89)
3-month follow-up	8/162 (4.94)	37/162 (22.84)	25/162 (15.43)	45/162 (27.78)	55/162 (33.95)
6-month follow-up	4/137 (2.92)	25/137 (18.25)	23/137 (16.79)	27/137 (19.71)	50/137 (36.50)
12-month follow-up	3/104 (2.88)	10/104 (9.62)	11/104 (10.58)	17/104 (16.35)	24/104 (23.08)

^a^Acute stress reaction was screened using Acute Stress Disorder Scales (ASDS) with the score for the dissociation cluster ≥9, combined with a total score of other clusters (ie, reexperiencing, avoidance, and arousal) ≥28.

^b^The posttraumatic stress disorder was measured by Posttraumatic Stress Disorder Checklist for DSM-5 (PCL5). Probably cases were identified according to endorsing moderate or higher (≥2) for at least 1 intrusion symptom (items 1–5), 1 avoidance symptom (items 6–7), 2 negative alterations in cognitions and mood (items 8–14), and 2 hyperarousal symptoms (items 15–20), following the DSM-5 diagnostic guidelines.

^c^Patients who self-evaluated current severity above average were defined as who filled a score above the median score of baseline investigation: “Self-evaluated severity of present injury (scaled from 0 to 5, the median score is 4 in present study)”.

In July 2021, we have successfully extracted DNA and performed genechip sequencing for 1,168 out of 1,173 delivered (99.6%) blood cell samples. The DNA quality control and genotyping were carried out at the WeGene Clinical Laboratory, Shenzhen. Genotyping was performed on the Illumina Infinium Chinese Genotyping Array BeadChip: Illumina WeGene V3 Arrays (∼700k variants). In addition, we used 539 feces samples for 16s rDNA sequencing (successful rate = 99.3%, 535/539, Lc-Bio Technologies Co., Ltd, Hangzhou, China), and the data cleaning for these sequenced data has been done. These abovementioned efforts demonstrated high quality of those collected bio-samples and the feasibility of our planned sequencing pipelines.

## STRENGTHS AND LIMITATIONS

To the best of our knowledge, this is the first hospital-based cohort study focusing on trauma-related psychiatric and physical outcomes among the Chinese population, with enriched data about a wide range of environmental and biological components, as well as lifetime trauma experience and medical care received after trauma exposure. The major merit of the CSTC includes a reasonably high baseline response rate (87.1%), as well as frequent and successful active follow-ups within 12 months after the participant recruitment (follow-up rates 76.5–90.0%), which largely attributed to a professional data collection team with four full-time workers and the well-customized EDC system (Cohort Data Management System, Version 1.0, Build 2021SR0484324. ©West China Hospital, Sichuan, China). In addition, our cohort applied multiple stringent quality control approaches for ensuring the high quality of collected data. Further, the multidimensional data merged from different data sources—trauma experience, lifestyle factors, somatic and psychiatric history from designed questionnaires, process of medical management and laboratory biomarkers from periodically linked data from EMR system, and biological data generated by bio-sample tests (eg, genotyping and microbiota)—provide the potential of studying on the complex interplays of trauma-related, environmental, and biological components on interested health outcomes using this database.

Notable limitations of CSTC are the single center setting (ie, all participants were recruited from Trauma Center of West China Hospital). Despite being considered as a centralized trauma care in Sichuan, this study population has poor representativeness for either all traumatized individuals in the Sichuan Province or the whole of China. However, the data collection of CSTC is still ongoing, and we have plans to include more trauma centers, which could help improve the generalizability of findings based on this cohort. Second, although most of those used psychological scales have been widely used in community-based studies,^34^^–^^36^ and well-validated after translated to the Chinese version,^37^^–^^41^ analyses of our data gained some inconsistent results, compared to prior reports. Specifically, the incidence of acute stress reaction revealed by ASDS (7.8%) at baseline, as well as probable PTSD measured by PCL-5 during the whole study period (7.6% to 1.7%), was somehow lower than that reported by prior studies with similar hospital-based setting in China (12.1–51.0%)^42^^,^^43^ and other countries (10.4–53.5%).^44^^,^^45^ As the risk of reporting bias is low due to the face-to-face interview and audio checking conducted by trained data collectors, we consider the plausible explanations might be the relatively low trauma severity of our participants. This notion is supported by higher and more reasonable incidence rates of cases with severe stress reaction observed among patients experienced more severe traumatic events (ie, with above average perceived severity or experienced family/intimate friends/strangers died/injured in current event). In addition, trauma patients need urgent neurological treatment (eg, surgeries) or intensive care would not be admitted to trauma center and thereby cannot be included in our cohort, and despite the collection of enriched psychological-related information, it is difficulty to identify self-inflicted injuries among our participants. Future studies, aiming to verify these results, as well as develop more sensitive measurements to detect psychopathology (either new scales or customized cut-off points of these standard scales) and self-inflicted intentions among the local traumatized population, are highly warranted. Last, we failed to collect biological samples at each follow-up time point, which prevents us from identifying biomarkers that might be valuable in terms of monitoring the dynamic changes of these trauma-related outcomes.

## SUPPLEMENTARY MATERIAL

eTable 1. The detailed information of questionnaires and scales

eTable 2. Validity of applied scales among Chinese population

eTable 3. Trauma, somatic diseases, and psychiatric disorders history of study participants, overall and by different cause of trauma

eTable 4. Baseline characteristics of study participants who completed the planned follow-ups and those who did not at 1 month and 12 months

eTable 5. Incidence of psychopathology^a^ in the study participants
