# Supplementary material for: Cohort Profile: The China Severe Trauma Cohort (CSTC)
Source: J Epidemiol. 2024 Jan 5;34(1):41–50. doi: 10.2188/jea.JE20220290 (PMC10701251; doi:10.2188/jea.JE20220290)
Supplement: Supplementary file 1 [file je-34-041-s001.pdf]

eTable 1. The detailed information of questionnaires and scales

Category	Items	Option
**Customized questionnaires**		
Sociodemographic and life style factors	Age	Numeric variable
	Gender	(0) Female (1) Male
	Ethnics	Specifying
	Address	Specifying
	Living area	(1) Rural (2) Urban
	Duration of residence	Specifying
	Body weight (kg)	Numeric variable
	Height (m)	Numeric variable
	Education level	(0) Elementary school and below
		(1) Middle school
		(2) Senior high school or technical secondary school
		(3) College
		(4) Postgraduate and above
	Monthly household income (RMB)	(0) Less than 3000
		(1) 3000-6000
		(2) 6000-9000
		(3) 9000-12000
		(4) Above 12000
	Self-evaluated economic status	(0) Lower than average
		(1) Average
		(2) Above the average

	Occupation status	(0) Full-time<br>(1) Part-time<br>(2) Unemployed<br>(3) House-wife or house-husband<br>(4) Retired<br>(5) Student
	Marital status	(0) Married<br>(1) Unmarried<br>(2) Divorced<br>(3) Widowed
	Current smoking status	(0) No (1) Yes
	Current alcohol consumption status	(0) No (1) Yes
History of severe somatic diseases or psychiatric disorders	Have you ever suffered from cardiovascular or cerebrovascular diseases? (eg, hypertension, coronary heart disease, valvular disease)	(0) No (1) Yes
	If you chose "yes" in the previous question, what disease have you got?	(1) Hypertension<br>(2) Coronary heart disease<br>(3) Valvular disease<br>(4) Stroke or hemorrhage<br>(5) Others (specifying)
	Have you ever suffered from immune diseases? (eg, autoimmune thyroid disease, rheumatoid arthritis, inflammatory bowel disease)	(0) No (1) Yes

If you chose "yes" in the previous question, what disease have you got?	(1) Autoimmune thyroid diseases<br>(2) Rheumatoid arthritis<br>(3) Inflammatory bowel disease<br>(4) Psoriasis<br>(5) Systemic lupus erythematosus<br>(6) Others (specifying)
Have you ever suffered from metabolic diseases? (eg, obesity, diabetes, gout, hyperlipidemia, fatty liver)	(0) No (1) Yes
If you chose "yes" in the previous question, what disease have you got?	(1) Obesity<br>(2) Diabetes<br>(3) Gout<br>(4) Hyperlipidemia<br>(5) Fatty liver<br>(6) Others (specifying)
Have you ever suffered from neurological disorders? (eg, Parkinson's disease, Alzheimer's disease, epilepsy)	(0) No (1) Yes
If you chose "yes" in the previous question, what disease have you got?	(1) Parkinson's disease<br>(2) Alzheimer's disease<br>(3) Epilepsy<br>(4) Others (specifying)
Have you ever suffered from follow disease? If yes, please specify	(1) Deafness<br>(2) Blind<br>(3) Heart malformation<br>(4) Physical malformations

		(5) Blood system diseases<br>(6) Kidney diseases<br>(7) Neuromuscular diseases
	Did you have a history of brain injury with disturbance of consciousness (loss of consciousness)	(0) No (1) Yes
	Do you have a history of dependence/abuse of psychoactive substances? (eg, alcohol, ecstasy, cannabis, powder, cocaine, heroin)	(0) No (1) Yes
	Have you ever visited a psychiatric hospital/mental health center for psychiatric disorders?	(0) No (1) Yes
	Did you have chronic pain before the accident?	(0) No (1) Yes
Current trauma experience	Time passed since injury (months)	Specifying
	Time passed since injury (days)	Specifying
	The exact time of injury	Specifying
	The cause of present injury	(1) Traffic accident<br>(2) Explosion<br>(3) Falling<br>(4) Crushing<br>(5) Twisting

	(6) Spraining<br>(7) Cutting injury<br>(8) Other (specifying)
Do you feel the fear during the event?	(0) No (1) Yes
If yes, how would you assess the fear of your injury?	A score range from 0(mildest) to 7 (worst)
How would you evaluate the severity of present injury?	A score range from 0(mildest) to 5 (worst)
Do you have any family member, or intimidate friends hurt in present event?	(0) No (1) Yes
If yes, did you witness they been hurt?	(0) No (1) Yes
Do you have any family member, or intimidate friends died in present event?	(0) No (1) Yes
If yes, did you witness their death?	(0) No (1) Yes
Did any stranger hurt or died in present event?	(0) No (1) Yes
If yes, did you witness they been hurt or died?	(0) No (1) Yes
On this 10cm line, the pain level increases from left(0cm) to right(10cm). You currently feel your pain is at_cm?	Specifying

	Do you feel limitation of doing moderate physical activity (eg, moving the table, sweeping the floor, Tai chi, doing simple gymnastics)?	(1) Greatly<br>(2) Sometimes<br>(3) Never
	Do you feel limitation of walking up/down stairs [greatly, somehow, or never]	(1) Greatly<br>(2) Sometimes<br>(3) Never
	Do you ever loss of consciousness in present event?	(0) No (1) Yes
	If yes, how long have you lost your consciousness	Specifying (days or hours)
**Validated scales[a]**		
Pain	Pain Catastrophizing Scale [PCS]	The PCS is a 13-items [ranging from 0 (mildest) to 4 (worst)] instrument which measuring catastrophizing impacts (rumination, magnification, and helplessness) on pain experience. A total PCS score of 30 represents clinically relevant level of catastrophizing.
Independence	Functional Independence Measure [FIM]	FIM comprises 13 motor items and 5 cognitive items with a total scores range of 13-126, to evaluate the functional independence. A higher score on behalf of better independence.
Social Support	Multidimensional Scale of Perceived Social Support [MSPSS]	MSPSS is used to assess the perception of social support from friends, family, and significant others with 12-items [ranging from very strongly disagree (1) to very strongly agree (7)]. A total score 12-48 indicating low level of perceived social support, 49-68 indicating moderate level of perceived social support, and ≥69 indicating high level of perceived social support.

Prior exposure to traumatic events	Life Events Checklist [LEC]	LEC consists of 17 items inquiring about the experience of 16 common post-traumatic events and one item as an complement of which not mentioned before, and the responses to these items include experiencing, witnessing, and learning about it.
	Childhood Trauma Questionnaire-SF [CTQ-SF]	28 items (25 clinical items and three validity items of the CTQ-SF rated the frequency of adverse childhood experiences with response options range from "never true" (0) to "very often" (4). Higher scores on the abuse and neglect subscales reflect greater severity of childhood traumatic experiences. Items 2, 5, 7, 13, 19, 26 and 28 were reversed scored
Psychopathology after trauma	Acute Stress Disorder Scale [ASDS]	ASDS is a 19-items inventory which used to index acute stress disorder (ASD) and predicts posttraumatic stress disorder (PTSD). It scored on a 5-point scale that indicates the severity of ASD symptoms from 1 (not at all) to 5 (very much). A cutoff for the dissociation cluster⩾9 combined with a total score⩾28 for the reexperiencing, avoidance, and arousal clusters indicate probable ASD.
	Patient Health Questionnaire-9 [PHQ-9]	PHQ-9 is a 9-item instrument with each item scored from 0 (not at all) to 3 (nearly every day). A total score over than 5 but less than 10 indicated mild depression, while a score ⩾10 mean a moderate depression and above.
	Generalized Anxiety Disorder Scale [GAD-7]	GAD-7 is a brief and effective screener for anxiety. For 7 item ranges from 0 to 3, a higher score indicates a more severe symptom. A total score over than 5 but less than 10 indicated mild anxiety, while a score⩾10 mean a moderate anxiety and above.
	Posttraumatic Stress Disorder Checklist for DSM-5 [PCL-5]	PCL-5 is a 20-item inventory which assesses the presence and severity of PTSD symptoms. PCL-5 is a reliable and valid screening instrument in chinses population. Probably cases were identified according to endorsing moderate or higher (≥2) for at least 1 intrusion symptom (items 1-5), 1 avoidance symptom (items 6-7), 2 negative alterations in cognitions and mood (items 8-14), and 2

	hyperarousal symptoms (items 15-20), following the DSM-5 diagnostic guidelines.
Pittsburgh Sleep Quality Index [PSQI]	The 19-item PSQI assesses subjective sleep quality into seven dimensions: subjective sleep quality, sleep latency, sleep duration, habitual sleep efficiency, sleep disturbances, use of sleep medications, and daytime dysfunction. The score range for each item is 0 (no difficulty) to 3 (severe difficulty). A total score>5 represent significant sleep disturbance.

[a] All scales were well-validated Chinese edition.

eTable 2. Validity of applied scales among Chinese population

Scales	Reference	Cut off score (if any)	Reliability (Cronbach's α)	Validity (if any)
Pain Catastrophizing Scale (PCS)	Ximing Xu, et al. Pain Physician, 2015	-	0.91	CFA suggested 3-factor structure fitting best<br>● CMIN/DF: 1.68<br>● NNFI/CFI/GFI: >0.90<br>● RMSEA: <0.08
	Bangli Shen, et al. Mol Pain, 2018	-	0.87	CFA suggested a 3-factor structure fitting best<br>● Chi-square/df: 2.94<br>● NFI:0.86<br>● CFI:0.90<br>● RMSEA: 0.10
Functional Independence Measure (FIM)	Xiao-Xiao Wang, et al. Health Qual Life Outcomes, 2017	Not fully functional independence: a total score <126	0.93	CFA suggested a 5-factor structure fitting best, with an explained total variance of 40.42%
Multidimensional Scale of Perceived Social Support (MSPSS)	Dan Wang, et al. Psychol Res Behav Manag, 2021	-	0.95	CFA suggested a 3-factor structure fitting best<br>● Chi-square/df: 5.45<br>● NNFI/CFI/GFI: >0.90<br>● RMSEA: 0.10
	Kaina Zhou, et al. Compr Psychiatry, 2015	-	0.92	CFA suggested a 3-factor structure fitting best<br>● Chi-square/df: 8.39<br>● TLI/CFI/GFI/AGFI: >0.90

				● RMSEA: 0.11
Life Events Checklist (LEC)	Fulei Geng, et al. J Affect Disord, 2021	Experienced: score of each item ≤4	0.85	-
Childhood Trauma Questionnaire-SF (CTQ-SF)	Wen-Juan Jiang, et al. PLoS One, 2018	Low to moderate: EA score≤12, EN score≤14, PN score≤9, PA score≤9, SA score≤7<br>Moderate to severe: EA score > 12, EN score > 14, PN score > 9, PA score > 9, SA score > 7	0.81	CTQ-SF score was significantly correlated with the total CPMS score (r = 0.61).<br>Subscales of CTQ-SF and CPMS were also significantly correlated (abuse: 0.39–0.64; neglect: 0.22–0.43)
	Jiayue He, et al. Child Abuse Negl, 2019	-	0.79	CFA suggested a 5-factor structure in two groups fitting best<br>● CMIN/DF for undergraduate sample:4.92<br>● CMIN/DF for depression sample:1.34<br>● TLI/CFI: >0.90<br>● RMSEA: <0.05
Acute stress disorder scale (ASDS)	Li Wang, et al. Pers Individ Dif, 2010	Score of dissociation cluster≥9 combined with a total score≥28 for the reexperiencing, avoidance, and arousal clusters	0.92	CFA suggested a 4-factor structure in two groups<br>● CMIN/DF:1.48<br>● AIC:490.94<br>● TLI/CFI: >0.99<br>● RMSEA: 0.04
Pittsburgh Sleep	Chi Zhang, et al. Front Psychiatry, 2020	Poor sleep: a total	0.68	CFA suggested a 1-factor structure with 5

Quality Index (PSQI)		score≥7		components fitting best.<br>● Chi-square/df: 1.59<br>● AIC:490.94<br>● NFI/RFI/IFI/TLI/CFI: >0.93<br>● RMSEA: 0.03
	Dong-Qin Yan, et al. Front Psychiatry, 2021	Poor sleep: a total score > 5	0.72	Construct validity was established by significant relationships between PSQI and depression, anxiety, stress, and HRQoL (r≥0.36).
Patient Health Questionnaire-9 (PHQ-9)	Yue Sun, et al. BMC Psychiatry, 2020	Mild depression: a total score of 5–9<br>Moderate depression: a total score of 10–14<br>Moderately severe depression: a total score of 15–19<br>Severe depression: a total score≥20	0.89	PCA suggested a 2-factor structure fitting best, with an explained total variance of 54.41%<br>PHQ-9 total score showed a significant positive correlation with HAMD-17 total score (r = 0.61)
Generalized Anxiety Disorder Scale (GAD-7)	Xiaoyan He, et al. Shanghai Arch Psychiatry, 2010	Anxiety: a total score≥10	0.90	Construct validity was established by significant relationships between total score of GAD-7 and HADS, anxiety subscale of HADS, and HAMA (r≥0.66)<br>● Sensitivity:0.86<br>● Specificity:0.96
Posttraumatic Stress Disorder	Peng Cheng, et al. J Affect Disord, 2020	Provisional PTSD diagnosis: a total	0.91	CFA suggested a 7-factor structure model fitting best.

Checklist for DSM-5 (PCL-5)		score≥33		● Chi-square/df: 1.52<br>● TLI/CFI: >0.95<br>● RMSEA: 0.05<br>PCL-5 was significantly correlated PC-PTSD-5 (r = 0.44);
	Hong Wang Fung, et al. J Evid Based Soc Work,2019	Detected DSM-5 PTSD: a total score≥49	0.95	PCL-5 was also significantly correlated with the number of traumatic events as measured with the LEC-5 (r = 0.42), SRMH (r=-0.32) and the BPI-T20 (r = 0.64)<br>● Sensitivity:0.71<br>● Specificity:0.73

AIC, Akaike information criterion; AGFI, adjusted goodness-of-fit index; CFA, confirmatory factor analysis; CFI, comparative fitness index; CMIN/DF, a Satorra–Bentler scaled chi-square (S-Bχ2)/degrees of freedom(df) ratio; CPMS, Child Psychological Maltreatment Scale; GFI, goodness of fit index; HADS, Hospital Anxiety And Depression Scale; HAMA, Hamilton anxiety scale; HAMD-17, Hamilton depression scale-17; HRQoL, health-related quality of life; IFI, incremental fit index; NFI, normalized fit index; NNFI, non-normed fit index; PC-PTSD-5, Primary Care PTSD Screen for DSM-5; RFI, relative fit index; RMSEA, root mean square error of approximation; TLI, Tucker-Lewis index.

eTable 3. Trauma, somatic diseases, and psychiatric disorders history of study participants, overall and by different cause of trauma

Variable	Total[a] N=2,500	By cause of trauma		
		fall/wrench N=1,190	traffic accident N=654	other N=644
**Trauma history**				
Lifetime trauma experience[b]				
As continuous variable, mean (SD)	2.55 (2.12)	2.35 (2.24)	2.81 (1.87)	2.66 (2.09)
By number of events				
0–1	864 (34.56)	504 (42.35)	149 (22.78)	209 (32.45)
2–3	1049 (41.96)	454 (38.15)	332 (50.76)	254 (39.44)
≥4	587 (23.48)	232 (19.50)	173 (26.45)	181 (28.11)
Childhood trauma experience[c], mean (SD)				
Total score	34.14 (7.59)	34.17 (7.81)	33.79 (6.96)	34.49 (7.8)
Emotional neglect	9.26 (3.72)	9.38 (3.80)	9.05 (3.5)	9.26 (3.81)
Physical neglect	8.58 (3.37)	8.57 (3.42)	8.49 (3.23)	8.70 (3.41)
Emotional abuse	5.85 (1.74)	5.77 (1.73)	5.84 (1.67)	6.00 (1.81)
Physical abuse	5.42 (1.26)	5.41 (1.27)	5.37 (1.03)	5.49 (1.43)
Sex abuse	5.04 (0.38)	5.03 (0.34)	5.04 (0.47)	5.04 (0.35)
**Somatic diseases or psychiatric disorders history**				
History of cerebro-cardiovascular diseases, n (%)				
No	2,227 (89.08)	1,028 (86.39)	593 (90.67)	603 (93.63)
Yes	199 (7.96)	129 (10.84)	40 (6.12)	30 (4.66)
Unknown	74 (2.96)	33 (2.77)	21 (3.21)	11 (1.71)
History of immune diseases, n (%)				
No	2,412 (96.48)	1,148 (96.47)	631 (96.48)	630 (97.83)
Yes	14 (0.56)	9 (0.76)	2 (0.31)	3 (0.47)
Unknown	74 (2.96)	33 (2.77)	21 (3.21)	11 (1.71)
History of metabolic diseases, n (%)				
No	2,269 (90.76)	1,053 (88.49)	595 (90.98)	618 (95.96)
Yes	157 (6.28)	104 (8.74)	38 (5.81)	15 (2.33)
Unknown	74 (2.96)	33 (2.77)	21 (3.21)	11 (1.71)
History of neurological disorders, n (%)				

No	2,451 (98.04)	1,161 (97.56)	648 (99.08)	638 (99.07)
Yes	30 (1.20)	22 (1.85)	4 (0.61)	4 (0.62)
Unknown	19 (0.76)	7 (0.59)	2 (0.31)	2 (0.31)
History of other somatic disease, n (%)				
No	2,354 (94.16)	1,119 (94.03)	615 (94.04)	617 (95.81)
Yes	72 (2.88)	38 (3.19)	18 (2.75)	16 (2.48)
Unknown	74 (2.96)	33 (2.77)	21 (3.21)	11 (1.71)
History of psychiatric disorder, n (%)				
None	2,411 (96.44)	1,138 (95.63)	638 (97.55)	624 (96.89)
Anxiety	40 (1.60)	26 (2.18)	7 (1.07)	7 (1.09)
Depression	25 (1.00)	11 (0.92)	7 (1.07)	7 (1.09)
Sleep disturbance	9 (0.36)	7 (0.59)	2 (0.31)	0 (0.00)
Other	11 (0.44)	5 (0.42)	0 (0.00)	6 (0.93)
Unknown	4 (0.16)	3 (0.25)	0 (0.00)	0(0.00)
History of psychoactive substance dependence, n (%)				
No	2,440 (97.60)	1,168 (98.15)	637 (97.40)	624 (96.89)
Yes	56 (2.24)	20 (1.68)	16 (2.45)	20 (3.11)
Unknown	4 (0.16)	2 (0.17)	1 (0.15)	0 (0.00)
Having chronic pain before the accident, n (%)				
No	2,074 (82.96)	952 (80.00)	562 (85.93)	550 (85.40)
Yes	421 (16.84)	234 (19.66)	92 (14.07)	94 (14.60)
Unknown	5 (0.20)	4 (0.34)	0 (0.00)	0 (0.00)

[a] 12 participants with missing value of the trauma causes were not shown in these table.

[b] Lifetime trauma experience was measured by Life Events Checklist (LEC). Participants have trauma exposure were defined as they directly experienced, or witnessed, or heard of trauma events.

[c] Childhood trauma experience was measured using Childhood Trauma Questionnaire-SF [CTQ-SF].

eTable 4. Baseline characteristics of study participants who completed the planned follow-ups and those who did not at 1 month and 12 months

Characteristic	1-month		12-month	
	Completed N=2,169 (90.00)	Loss of follow-up N=241 (10.00)	Completed N=1,235 (89.04)	Loss of follow-up N=152 (10.96)
Male, n (%)	1357 (62.56)	152 (63.07)	793 (64.21)	108 (71.05)
Age, Mean (SD), year	45.77 (16.42)	47.62 (16.42)	45.54 (16.42)	45.91 (15.36)
Body mass index, Mean (SD), kg/m^2	23.37 (3.45)	23.31 (3.54)	23.31 (3.39)	23.54 (3.58)
<18.5	158 (7.28)	16 (6.64)	91 (7.37)	7 (4.61)
18.5–25	1317 (60.72)	142 (58.92)	759 (61.46)	93 (61.18)
25–30	565 (26.05)	59 (24.48)	295 (23.89)	43 (28.29)
≥30	76 (3.50)	9 (3.73)	43 (3.48)	6 (3.95)
Unknown	53 (2.44)	15 (6.22)	47 (3.81)	3 (1.97)
Region, n (%)				
Urban	842 (38.82)	87 (36.10)	497 (40.24)	56 (36.84)
Rural	1294 (59.66)	141 (58.51)	705 (57.09)	91 (59.87)
Unknown	33 (1.52)	13 (5.39)	33 (2.67)	5 (3.29)
Current smoking status[a], n (%)				
No	1794 (82.71)	197 (81.74)	1019 (82.51)	128 (84.21)
Yes	372 (17.15)	38 (15.77)	213 (17.25)	24 (15.79)
Unknown	3 (0.14)	6 (2.49)	3 (0.24)	0 (0.00)
Current alcohol consumption[b], n (%)				
No	1774 (81.79)	209 (86.72)	1060 (85.83)	126 (82.89)
Yes	393 (18.12)	26 (10.79)	173 (14.01)	26 (17.11)
Unknown	2 (0.09)	6 (2.49)	2 (0.16)	0 (0.00)
Marital status, n (%)				
Unmarried	422 (19.46)	30 (12.45)	239 (19.35)	26 (17.11)
Married	1631 (75.20)	184 (76.35)	916 (74.17)	117 (76.97)
Divorced/Widowed	111 (5.12)	21 (8.71)	75 (6.07)	9 (5.92)
Unknown	5 (0.23)	6 (2.49)	5 (0.40)	0 (0.00)
Education, n (%)				
Elementary and lower	428 (19.73)	49 (20.33)	246 (19.92)	30 (19.74)
Junior school	657 (30.29)	73 (30.29)	389 (31.50)	41 (26.97)
Senior/Secondary school	526 (24.25)	58 (24.07)	288 (23.32)	35 (23.03)
Collage and above	442 (20.38)	50 (20.75)	202 (16.36)	35 (23.03)
Unknown	116 (5.35)	11 (4.56)	110 (8.91)	11 (7.24)
Occupation, n (%)				
Full-time	1296 (59.75)	139 (57.68)	707 (57.25)	104 (68.42)
Part-time or unemployed[c]	490 (22.59)	50 (20.75)	324 (26.23)	29 (19.08)

Retired	383 (17.66)	52 (21.58)	204 (16.52)	19 (12.50)
Self-evaluated economic status, n (%)				
<average	315 (14.52)	48 (19.92)	208 (16.84)	33 (21.71)
average	1615 (74.46)	147 (61.00)	815 (65.99)	103 (67.76)
>average	104 (4.79)	25 (10.37)	67 (5.43)	10 (6.58)
Unknown	135 (6.22)	21 (8.71)	145 (11.74)	6 (3.95)
Monthly household income (RMB), n (%)				
<3,000	448 (20.65)	42 (17.43)	291 (23.56)	42 (27.63)
3,000–6,000	575 (26.51)	72 (29.88)	267 (21.62)	40 (26.32)
6,000–9,000	380 (17.52)	40 (16.60)	181 (14.66)	23 (15.13)
⩾9,000	565 (26.05)	61 (25.31)	286 (23.16)	37 (24.34)
Unknown	201 (9.27)	26 (10.79)	210 (17.00)	10 (6.58)
Perceived social support[d], n (%)				
Low supported	56 (2.58)	0 (0.00)	35 (2.83)	3 (1.97)
Moderate supported	361 (16.64)	0 (0.00)	228 (18.46)	30 (19.74)
High supported	1732 (79.85)	0 (0.00)	851 (68.91)	85 (55.92)
Unknown	20 (0.92)	241 (100.00)	121 (9.80)	34 (22.37)
History of cerebro-cardiovascular diseases, n (%)				
No	1941 (89.49)	213 (88.38)	1141 (92.39)	145 (95.39)
Yes	168 (7.75)	16 (6.64)	87 (7.04)	7 (4.61)
Unknown	60 (2.77)	12 (4.98)	7 (0.57)	0 (0.00)
History of immune diseases, n (%)				
No	2099 (96.77)	228 (94.61)	1223 (99.03)	152 (100.00)
Yes	10 (0.46)	1 (0.41)	5 (0.40)	0 (0.00)
Unknown				
History of metabolic diseases, n (%)	60 (2.77)	12 (4.98)	7 (0.57)	0 (0.00)
No	1980 (91.29)	217 (90.04)	1153 (93.36)	144 (94.74)
Yes	129 (5.95)	12 (4.98)	75 (6.07)	8 (5.26)
Unknown	60 (2.77)	12 (4.98)	7 (0.57)	0 (0.00)
History of neurological disorders, n (%)				
No	2134 (98.39)	231 (95.85)	1205 (97.57)	149 (98.03)
Yes	27 (1.24)	1 (0.41)	21 (1.70)	1 (0.66)
Unknown	8 (0.37)	9 (3.73)	9 (0.73)	2 (1.32)
History of other somatic disease, n (%)				
No	2059 (94.93)	219 (90.87)	1193 (96.60)	149 (98.03)
Yes	50 (2.31)	10 (4.15)	35 (2.83)	3 (1.97)

Unknown	60 (2.77)	12 (4.98)	7 (0.57)	0 (0.00)
History of psychiatric disorder, n (%)				
None	2099 (96.77)	226 (93.78)	1202 (97.33)	147 (96.71)
Anxiety	35 (1.61)	3 (1.24)	9 (0.73)	2 (1.32)
Depression	20 (0.92)	3 (1.24)	14 (1.13)	3 (1.97)
Sleep disturbance	7 (0.32)	2 (0.83)	5 (0.40)	0 (0.00)
Other	8 (0.37)	3 (1.24)	4 (0.32)	0 (0.00)
Unknown	0 (0.00)	4 (1.66)	1 (0.08)	0 (0.00)
History of psychoactive substance dependence, n (%)				
No	2122 (97.83)	230 (95.44)	1208 (97.81)	151 (99.34)
Yes	47 (2.17)	7 (2.90)	25 (2.02)	1 (0.66)
Unknown	0 (0.00)	4 (1.66)	2 (0.16)	0 (0.00)
Having chronic pain before the accident, n (%)				
No	1802 (83.08)	195 (80.91)	1050 (85.02)	132 (86.84)
Yes	366 (16.87)	42 (17.43)	183 (14.82)	20 (13.16)
Unknown	1 (0.05)	4 (1.66)	2 (0.16)	0 (0.00)

[a] Participant chose “YES” in “Smoking” item was defined as current smoker

[b] Participant chose “YES” in “Drinking alcohol” item was defined as current alcohol drinker

[c] “unemployed” including the unemployed, housewife, house-husband, and student

[d] Perceived social support was assessed by Multidimensional Scale of Perceived Social Support [MSPSS], with a total score 12–48 indicating low level of perceived social support, 49–68 indicating moderate level of perceived social support, and ≥69 indicating high level of perceived social support.

eTable 5. Incidence of psychopathology[a] in the study participants

Type of psychopathology	Completeness[b] n (%)	Without symptoms n (%)	With symptoms, n (%)		
			Any	Mild	Moderate/severe symptom
Depression[c]					
Baseline	908/911 (99.67)	703 (77.42)	205 (22.58)	152 (16.74)	53 (5.84)
1-month follow-up	2,159/2,410 (89.59)	1,818 (84.21)	341 (15.79)	241 (11.16)	100 (4.63)
3-month follow-up	1,682/2,213 (76.01)	1,501 (89.24)	181 (10.76)	123 (7.31)	58 (3.45)
6-month follow-up	1,452/1,920 (75.62)	1,295 (89.19)	157 (10.81)	109 (7.51)	48 (3.31)
12-month follow-up	1,227/1,387 (88.46)	1,115 (90.87)	112 (9.13)	89 (7.25)	23 (1.87)
Anxiety[d]					
Baseline	908/911 (99.67)	743 (81.83)	165 (18.17)	128 (14.10)	37 (4.07)
1-month follow-up	2,159/2,410 (89.59)	1,885 (87.31)	274 (12.69)	193 (8.94)	81 (3.75)
3-month follow-up	1,684/2,213 (76.10)	1,517 (90.08)	167 (9.92)	122 (7.24)	45 (2.67)
6-month follow-up	1,454/1,920 (75.73)	1,321 (90.85)	133 (9.15)	91 (6.26)	42 (2.89)
12-month follow-up	1,231/1,387 (88.75)	1,153 (93.66)	78 (6.34)	58 (4.71)	20 (1.62)
Sleep disturbance[e]					
Baseline	2,499/2,500 (99.96)	1,514 (60.58)	985 (39.42)	-	-
1-month follow-up	2,161/2,410 (89.67)	1,301 (60.20)	860 (39.80)	-	-
3-month follow-up	1,685/2,213 (76.14)	1,128 (66.94)	557 (33.06)	-	-
6-month follow-up	1,454/1,920 (75.73)	1,029 (70.77)	425 (29.23)	-	-
12-month follow-up	1,233/1,387 (88.90)	928 (75.26)	305 (24.74)	-	-

[a] The presence of psychopathology were defined as participants have any positive symptoms regarding anxiety, depression, or sleep quality.

[b] Completeness was calculated by dividing the number of participants who finished this scale by that who were supposed to answer this scale.

[c] Depression symptoms were measure by Patient Health Questionnaire-9 (PHQ-9), with a total score 5–9 indicating mild depression, and ⩾10 for moderate/severe depression.

[d] Anxiety symptoms were measure by Generalized Anxiety Disorder Scale (GAD-7), with a total score 5–9 indicating mild anxiety, and ⩾10 for moderate/severe anxiety.

[e] Significant sleep disturbance were determined by Pittsburgh Sleep Quality Index (PSQI) with a cut-off score >5 for considerable sleep problems.
